# Genome-Wide Investigation of the NAC Transcription Factor Family in *Miscanthus sinensis* and Expression Analysis Under Various Abiotic Stresses

**DOI:** 10.3389/fpls.2021.766550

**Published:** 2021-11-04

**Authors:** Gang Nie, Zhongfu Yang, Jie He, Aiyu Liu, Jiayi Chen, Shuan Wang, Xia Wang, Guangyan Feng, Dandan Li, Yan Peng, Linkai Huang, Xinquan Zhang

**Affiliations:** Department of Forage Science, College of Grassland Science and Technology, Sichuan Agricultural University, Chengdu, China

**Keywords:** *Miscanthus sinensis*, NAC transcription factor, gene family, abiotic stress, gene expression

## Abstract

The NAC transcription factor family is deemed to be a large plant-specific gene family that plays important roles in plant development and stress response. *Miscanthus sinensis* is commonly planted in vast areas of marginal lang as forage, ornamental grass, or bioenergy crop which demand a relatively high resistance to abiotic stresses. The recent release of a draft chromosome-scale assembly genome of *M. sinensis* provided a basic platform for the genome-wide investigation of NAC proteins. In this study, a total of 261 *M. sinensis NAC* genes were identified and a complete overview of the gene family was presented, including gene structure, conserved motif compositions, chromosomal distribution, and gene duplications. Results showed that gene length, molecular weights (MW), and theoretical isoelectric points (pI) of NAC family were varied, while gene structure and motifs were relatively conserved. Chromosomal mapping analysis found that the *M. sinensis NAC* genes were unevenly distributed on 19 *M. sinensis* chromosomes, and the interchromosomal evolutionary analysis showed that nine pairs of tandem duplicate genes and 121 segmental duplications were identified, suggesting that gene duplication, especially segmental duplication, is possibly associated with the amplification of *M. sinensis NAC* gene family. The expression patterns of 14 genes from *M. sinensis* SNAC subgroup were analyzed under high salinity, PEG, and heavy metals, and multiple *NAC* genes could be induced by the treatment. These results will provide a very useful reference for follow-up study of the functional characteristics of *NAC* genes in the mechanism of stress-response and potential roles in the development of *M. sinensis*.

## Introduction

*Miscanthus sinensis* is a rhizomatous C4 perennial grass which is widely applied in paper production and cultivated as forage, ornamental grass, and a bioenergy crop (Clifton-Brown and Lewandowski, 2002; Heaton et al., 2004; Nie et al., 2021). Generally, *M. sinensis* tend to be planted in marginal land with varied kinds of abiotic stress, such as limited water supply and heavy metal pollution (Yu et al., 2014), so as to maximize the avoidance competition of arable land (Mohr and Raman, 2013). *Miscanthus sinensis* can produce high biomass yields and potentially increase soil carbon when compared with other C4 grasses like *Zea mays* and *Saccharum officinarum*, which is enabled by a relatively highly efficient photosynthesis process and a great resistance to abiotic stress (Greef et al., 1997; Ye et al., 2005; Ezaki et al., 2008; Long and Spence, 2013; Nie et al., 2017) owing to its extensive root system and perennial growth habit (Byrt et al., 2011; Van der Weijde et al., 2013). However, the molecular regulation network and genetic components of the characteristics are still unclear due to the absence of genomic information of the species owing to the complexity of its genomes, which hinders the application of modern breeding approaches (Mitros et al., 2020).

Continued genetic improvement of bioenergy plants for their excellent features is important, especially to enhance productivity and resistance when exposed to abiotic stress. Recently, a draft chromosome-scale assembly of *M. sinensis* genome was published, which promoted the process of future genetic improvement of perennial biomass crops (Mitros et al., 2020). NAC proteins have shown significant functions in controlling plant growth and development in active response to abiotic and/or biotic stress (Aida et al., 1997; Nakashima et al., 2012; Kou et al., 2014). NAC domain protein family is deemed a transcription factor (TF) family containing a highly conserved N-terminal DNA-binding domain and a variable C-terminal domain. Commonly, it is divided into five subdomains (A–E) and was originally identified from three consensus sequences: NAM (no apical meristem), *Arabidopsis* ATAF1/2, and CUC2 (cup-shaped cotyledon; Ooka et al., 2003; Nakashima et al., 2007; Jensen et al., 2010). Although *NAC* gene family has been widely studied and the identified genes could potentially be used for plant improvement, there is no systematic concern in *M. sinensis* referring to limited availability of genome resources.

In *Arabidopsis*, *AtNAC1* and *AtNAC2* were reported to be involved in lateral root development by downregulating auxin signals (Guo et al., 2005), while *NAP* is related to plant leaf senescence (Guo and Gan, 2006) and floral morphogenesis (Sablowski and Meyerowitz, 1998). In *Medicago truncatula*, loss of *MtNST1* resulted in the decrease of lignin content (Zhao et al., 2010). The overexpression of *TaNAC69* enhanced the transcript levels of stress-induced genes and improved the drought tolerance of *Triticum aestivum* (Xue et al., 2011), while the overexpression of *TaNAC29* increased salt tolerance by reducing H_2_O_2_ accumulation and membrane damage (Xu et al., 2015). *OsNAC6* was induced by low temperature, salinity, drought, and ABA damage (Ohnishi et al., 2005; Nakashima et al., 2007). In soybean (*Glycine max*), 31 genes were identified to contain complete open reading frames encoding GmNAC protein. Among them, nine of the *GmNAC* genes were induced by drought, salinity, cold, and ABA treatments (Tran et al., 2009). In addition, the *SsNAC23* gene in sugarcane homologous to *OsNAC6* is associated with cold and PEG-mediated water stress (Nogueira et al., 2005). In Tartary buckwheat (*Fagopyrum tataricum*), eight *FtNAC* genes were identified as potentially participating in fruit development (Liu et al., 2019). In addition, stress-responsive NAC (SNAC) proteins were also reported to have critical roles in regulating plant abiotic stress tolerance, which would be useful for further genetic improvement (Nakashima et al., 2012).

The genome-wide identification and analysis of the *NAC* gene family will provide an important foundation to understand how abiotic stress response and signaling has evolved in plant development. In this study, a complete overview was reported on gene structure, duplication, and phylogenetic characteristics of *M. sinensis NAC* genes at the genome level. Also, an expression analysis of *NAC* genes that suffered from various abiotic stresses was comprehensively analyzed. The result will be helpful for further study of the functional characteristics of *NAC* genes in the mechanism of *M. sinensis* stress-responsive.

## Materials and Methods

### Identification of NAC Genes in *M. sinensis*

The *M. sinensis* genome resource was available in the PHYTOZOME database^1^ (Mitros et al., 2020). The HMM profile for the NAC transcription factor (PF02365) was downloaded from the Pfam database^2^ (Eddy, 2011). The NAC proteins from *M. sinensis* genome were identified from the annotated protein database and were used by HMMER 3.0 software (default parameters) on the similarity in sequence network with Hidden Markov Model (the cutoff value was set to 0.01; Finn et al., 2014; Lozano et al., 2015; Moturu et al., 2018). Then, the candidate *NAC* genes were further confirmed by Pfam and NCBI CDD. All candidate protein sequences through the batch sequence search of Pfam^3^ were uploaded to search for domain NAM structure (default parameters). Similarly, we uploaded all candidate protein sequences to NCBI batch CD-search^4^ for the structural domain search. Finally, the purified protein model^5^ was used to analyze the physicochemical parameters, including the CDs (Coding Sequence) length, molecular weight (MW), and theoretical isoelectric points (PI; Wilkins et al., 1999). Cis-acting regulatory elements were analyzed using PlantCARE software (Lescot et al., 2002). The online ProtComp 9.0^6^ was used to predict the subcellular localization of the protein. The membrane-bound NAC proteins were predicted by TMHMM server v.2.0^7^ (Zhou et al., 2018b).

### Phylogenetic Analysis and Classification of the *M. sinensis* NAC Gene Family

The NAC protein sequences of *Arabidopsis* were retrieved from the TAIR 11 (*Arabidopsis* genome database; Rhee et al., 2003).^8^ The amino acid sequence alignment of *M. sinensis* and *Arabidopsis thaliana NAC* genes was performed by the combination of MEGA version 6.0 and Genedoc software (Nicholas et al., 1997), and the phylogenetic tree was constructed by using the MEGA 6.0 with Poisson model, using the NJ method with pairwise gaps deletion and 1,000 bootstraps (Tamura et al., 2011). According to the classification of AtNAC, the 261 *M. sinensis NAC* genes were divided into different subgroups.

### Gene Structure and Motif Analysis

To explore the exon/intron structure of *M. sinensis NAC* genes, the online Gene Structure Display Server 2.0 tools was used. The conserved motif of NAC proteins of *M. sinensis* was predicted by using the Multiple Em for Motif Elicitation (MEME)^9^ program with the default settings (Bailey et al., 2015).

### Chromosomal Mapping, Gene Replication, and Syntenic Analysis With Several Plant Species

The chromosomal positions information of *NAC* genes was acquired from the *M. sinensis* genome annotations. The chromosomal map of *M. sinensis NAC* genes was drafted by MG2C.^10^ Segmental and tandem duplications have been suggested to represent two of the main causes of gene family expansion in plants (Cannon et al., 2004). Segmental duplications of multiple genes through polyploidy followed by chromosome rearrangements were carried out (Yu et al., 2005). Tandem duplications were characterized as multiple members of one family occurring within the same intergenic region or in neighboring intergenic regions (Ramamoorthy et al., 2008). In this study, MCScanX (default parameters) was used to examine segmental and tandem duplications (Wang et al., 2012). The corresponding plot was created by Dual Synteny Plot for MCscanx in TBtools platform^11^ (Chen et al., 2020), and the *NAC* gene ID of *M. sinensis* was highlighted to obtain homologous pairs. Thus, the syntenic analysis maps between *M. sinensis* and other 10 plants (*Brachypodium distachyon*, *Panicum hallii*, *Saccharum spontaneum*, *Setaria italic*, *Sorghum bicolor*, *Z. mays*, *M. truncatula*, *G. max*, *Trifolium repens*, and *A. thaliana*) were constructed.

### Plant Materials, Growth Conditions, and Stress Treatments

The *M. sinensis* plant seeds “0819” were collected from 29°57′30.1″, 102°24′36.4″, and 1,388 m asl. The plastic pots with a matrix of quartz sand were used to plant the *M. sinensis* seedlings, and 1X Hoagland’s nutrient solution was used to cultivate. Plant materials were grown under a constant temperature (28/25°C with cycles of 12/12 h for light and darkness), respectively, in the incubator. When the plants had 5–6 leaves, five different stress treatments started. These stress conditions were set to evaluate the gene expression pattern, including drought stress (20% PEG 6000), arsenic stress (As^5+^, 200 mg/L Na_2_HAsO_4_·7H_2_O), chromium stress (Cr^6+^, 200 mg/L K_2_Cr_2_O_7_), and cadmium stress (Cd^2+^, 200 mg/L CdCl_2_·2.5H_2_O), and salt stress (300 mmol/L NaCl). The plants were separately treated by five stresses for 6 days. At 0, 1, 3, and 6 days, samples under each treatment were obtained with three independent biological replicates. After that, all samples were immediately frozen in liquid nitrogen and then were kept at −80°C for RNA extraction.

### RNA Extraction, cDNA Synthesis, and qRT-PCR Gene Expression Analysis

Direct-zol™ RNA MiniPrep Kit (Zymo Research, China) was used to extract total RNA according to the manufacturer’s instructions. The genomic DNA was eliminated by DNase I (6 U/μl; Zymo Research, China). A NanoDrop ND-2000 (Thermo Scientific, United States) spectrophotometer and 1% agarose gel electrophoresis were used to detect the RNA quality of all samples. The cDNA was synthesized with The 5X All-In-One RT MasterMix (with AccuRT Genomic DNA Removal Kit) by following instructions, including total RNA 2 μg, 4X AccuRT Reaction Mix 2 μl, 5X AccuRT Reaction Stopper 2 μl, and 5X All-In-One RT MasterMix 4 μl. All cDNA was stored in reserve at −20°C. The qRT-PCR technique was performed to validate the expression of *M. sinensis NAC* genes selected from SNAC subgroup, and Premier 3.0 was used to design the specific primers (Table 1). A total of 10 μl volume consists of 5 μl abm® EvaGreen 2X qPCR Master Mix (Applied Biological Materials, Inc., Canada), 2.9 μl ddH_2_O, 1.5 μl synthesized cDNA product, and 0.3 μl each primer. Real-time RT-PCR reaction procedures are as follows: one cycle of 95°C for 10 min to activate enzyme, 95°C for 15 s for denaturation, and 60°C for 60 s for anneal/extension, for a total of 35 cycles. Finally, Tm and melting curve analysis were obtained to verify the specificity of each primer. The relative gene expression values were analyzed using the 2^−ΔΔCt^ method (Schmittgen and Livak, 2008). The reference gene (*Unigene33024*) was set to standardize the expression data (Zhong et al., 2020).

**Table 1:** Primers information for selected *M. sinensis* and reference genes.

Gene	Forward-primer (5′-3′)	Reverse-primer (5′-3′)	Subgroup
Misin05G308400.1	CACGAGGAACGGCATTG	GAACGGTGGCAGGATTGT	AtNAC3
Misin06G301900.1	CCGACCTGAATCTGGATG	CGGTGGCAGGATTGTCT	AtNAC3
Misin03G063600.1	TTCTACCACGGCAAGCCTCCTC	GCACAGCACCCAGTCATCCAAC	AtNAC3
Misin09G049100.1	GTTGCCGAGGACGGGTTCA	TGCAGGTAGGACGCGAGCAT	AtNAC3
MisinT507400.1	CAAGCCGCTCGCCATCAA	CAGCACCCAGTCATCCAACCT	ATAF
Misin06G349300.1	CCGCTCGCCATCAAGAAG	AGCACCCAGTCATCCAACCT	ATAF
Misin17G145400.1	CCCAAGGGCGACAAGACCA	TTCGGCACAGCACCCAATCA	ATAF
Misin16G144500.1	CCCAAGGGCGACAAGACCAA	GCACAGCACCCAATCATCCAAC	ATAF
Misin05G005800.1	CACCGACAAGCCCATCCTG	GACCCAGTCGTCAAGCCTCAT	NAP
Misin05G092200.1	ACTCGGCCATGTCGGAGCTGTT	CAGGGTCGCTGGTAACGAAGTAGAAC	NAP
Misin01G367800.1	CCCAAGGGCGACAAGACCAA	GCACCCAGTCATCCAGCCTCAT	NAP
Misin04G398400.1	CACCGACAAGCCCATCCACGAC	TGTAGATCCGGCAGAGCACCCAGT	NAP
Misin09G002600.1	AACAAGAGGAAACGGATGACC	GCTGTGCTGAAACAACCCAC	NAP
MisinT504500.1	ACAGCAAAGCCCTTCACTAATC	CACCGTTGTTATCTGAGCCTATT	NAP
Unigene33024	CCCGTTTGGTTCCACATCAA	CCGAACCTGCTGACTTTGTG	/

## Results

### Identification of the *M. sinensis NAC* Genes

To identify the putative NAC proteins in *M. sinensis* genome, a HMM (hidden Markov model) search was performed (NAM domain, PF02365) against the *M. sinensis* genome downloaded from Phytozome database.^1^ Subsequently, 261 putative *M. sinensis NAC* genes were identified after removing redundant sequences preliminarily. The protein sequence length of all *M. sinensis* NAC proteins ranged from 71 (*MisinT039500.1*) to 691 (*Misin18G098700.1*) amino acids with an average of 357. The molecular weights (MW) of the proteins ranged from 7575.7 to 77657.7 Da, and the theoretical isoelectric points (pI) ranged from 4.14 (*Misin14G114600.1*) to 10.88 (*Misin13G160800.1*), with an average of 6.92. The subcellular localization of 261 *M. sinensis* NAC proteins was predicted as nuclear (56.3%) or extracellular (43.7%). The basic information of all identified *M. sinensis NAC* genes, including the coding sequence length (CDs), protein sequence length, and genomic location, was mentioned in Supplementary Table S1. A further 12 *M. sinensis* NAC proteins were identified, which contained α-helical trans-membrane (TM) domain (Supplementary Table S1). They may respond to abiotic stress (Zhou et al., 2018b), and are highly homologous to *Arabidopsis* NAC2 and TIP, respectively.

### Phylogenetic Analysis and Classification of the *M. sinensis NAC* Gene Family

**Figure 1:**
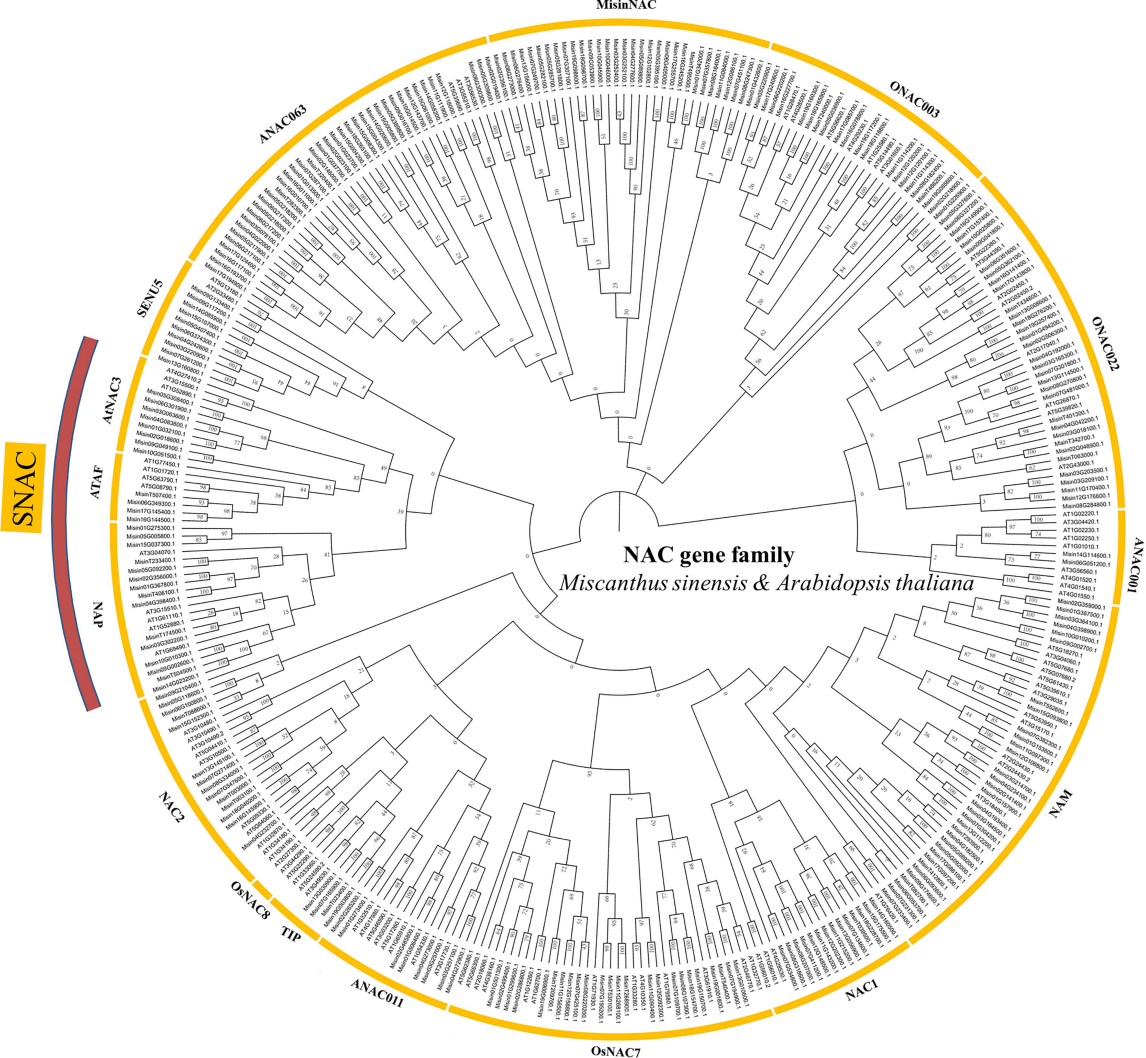
Unrooted phylogenetic tree representing relationships among the NAC proteins of *Miscanthus sinensis* and *Arabidopsis thaliana*. *NAC* genes were used to construct the Neighbor-Joining tree with the parameters including a Blosum62 cost matrix, the Jukes-Cantor model, global alignment, and bootstrap value of 1,000.

An unrooted neighbor-joining (NJ) tree (with 1,000 bootstraps) was constructed by using the amino acid sequence alignment of NAC proteins from *M. sinensis* and *A. thaliana* (Figure 1). The results showed that 261 *M. sinensis NAC* genes could be divided into 15 subgroups, ANAC063, SENU5, ONAC003, AtNAC3, ANAC001, ATAF, ANAC011, NAP, OsNAC7, NAC1, NAC2, ONAC022, TIP, NAM, and a *M. sinensis*-specific subgroup named MisinNAC together with *A. thaliana*. The ANAC001 subgroup contained only two *M. sinensis* NAC proteins, and the ANAC063 subgroup contained the most members of *M. sinensis* NAC proteins. However, no NAC members in *M. sinensis* were detected from the OsNAC8. Besides, many stress-responsive *NAC* genes that regulate gene expression associated with plant abiotic stress as factors were assigned to the SNAC subgroup, including AtNAC3, NAP, and ATAF in the present study. Cis-elements analysis of *SNAC* genes in *M. sinensis* showed that most of genes may be associated in a variety of plant stress response pathways and hormonal regulation, such as auxin, abscisic acid, and MeJA (Supplementary Table S2).

### Conserved Motif and Gene Structure Analysis of *M. sinensis NAC* Genes

**Figure 2:**
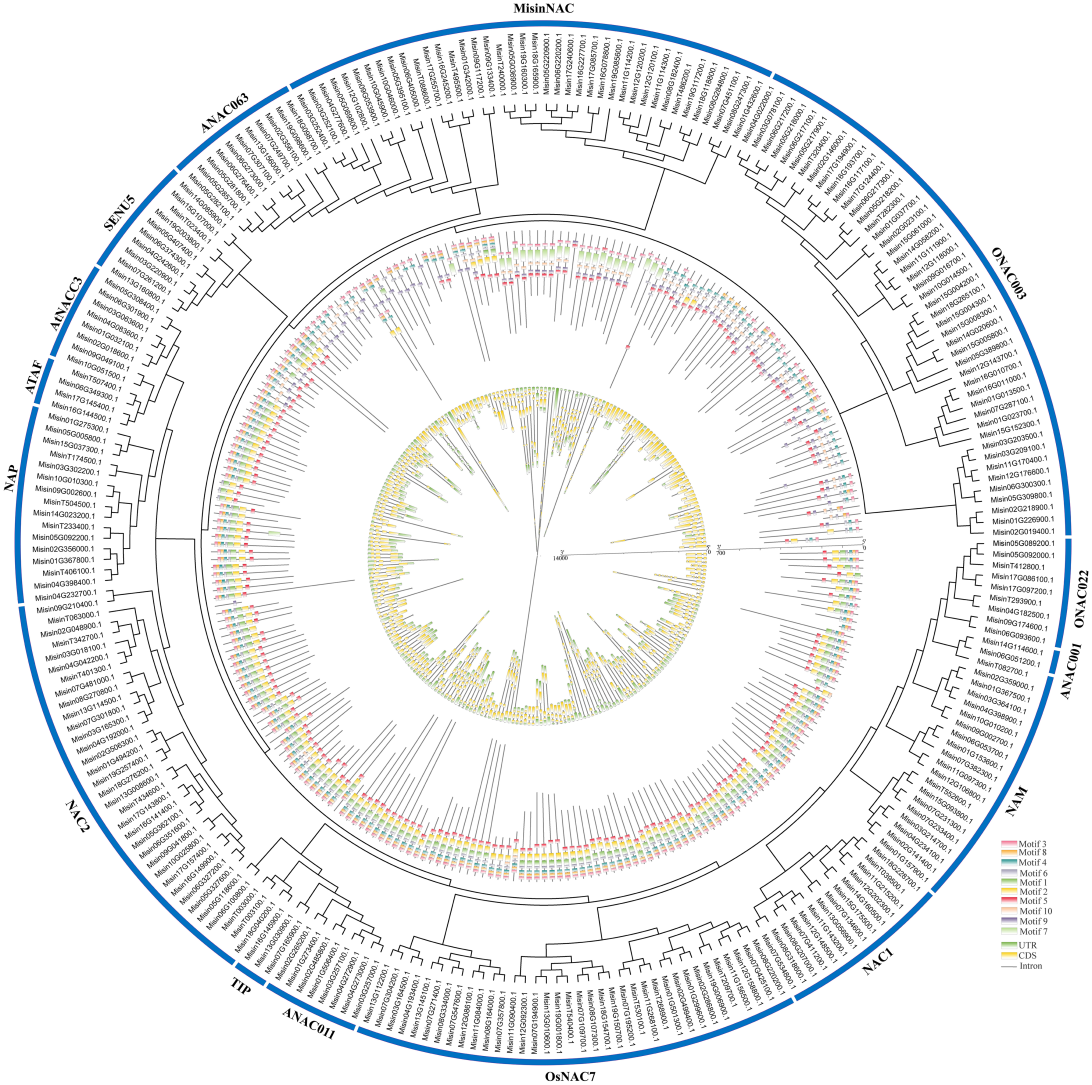
Phylogenetic relationships, motifs pattern, and gene structure of *NAC* genes from *M. sinensis*. The unrooted phylogenetic tree was constructed based on the full-length sequences of NAC proteins. The motifs composition of the NAC proteins was identified by MEME, and grey lines represent the non-conserved sequences. Each motif is indicated by a colored box numbered at the bottom, and the sequence information for each motif is provided in Supplementary Figure S1. The exon-intron structure of *NAC* genes was performed by using the online tool GSDS. Lengths of exons and introns of each *M. sinensis NAC* gene were exhibited proportionally.

Ten conserved motifs were identified in the MEME tool to reveal the structural similarity and diversification of *M. sinensis* NAC proteins (Figure 2). The length of these conserved motifs was varied (11–57 amino acids) and showed a highly diverse distribution. And the details of the sequence logos of each motif were listed in Supplementary Figure S1. *Misin10G045900.1* only contains one motif, whereas eight types motifs were present in *Misin19G098600.1* (which contained the highest number). In the NAC2, AtNAC3, ATAF, and NAP subgroups, motif 1–6 and motif 8 were the most conserved parts. Motif 10 only appeared in ONAC003, ANAC063, SENU5, and MisinNAC, which may reveal that it was a typical part of these subgroups. Similar motif compositions and similar positions were exhibited in *M. sinensis NAC* genes (which located in the same subgroup), indicating that genes in the same subgroup may have similar functions. However, the specific biological function of most of these motifs is not classified and requires further investigation. The structural features of all identified *NAC* genes in *M. sinensis* were analyzed to estimate the evolution of the *NAC* gene family. As shown in Figure 2, among the coding sequences of the entire *NAC* genes, 18 (approximately 6.90%) were intronless, and over half (132, approximately 50.57%) had three exons. Only three genes (*Misin11G114300.1*, *Misin09G174600.1*, and *Misin01G432600.1*) had more than seven exons. Most genes from the same subgroups had a similar exon/intron structure, and *Misin18G098700.1* had more than 20 kb gene structure, while *MisinT039500.1* had the shortest structure with 216 bp.

### Chromosomal Locations and Synteny Analysis of *M. sinensis NAC* Genes

**Figure 3:**
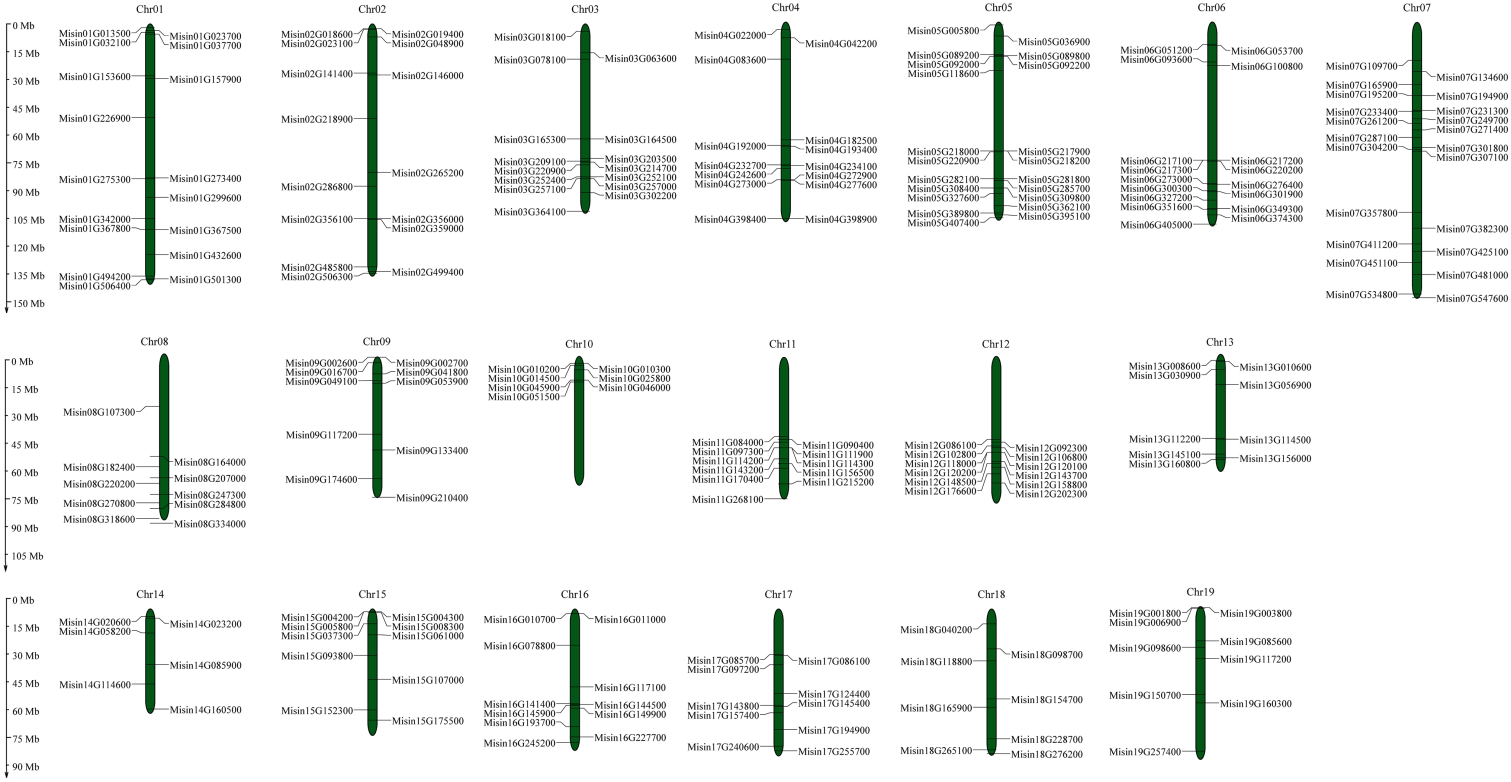
Distribution of 234 *M. sinensis NAC* genes on 19 chromosomes. Vertical bars represent the chromosomes, and the chromosome number is to the top of each chromosome. The scale on the left represents chromosome length (Mb).

**Figure 4:**
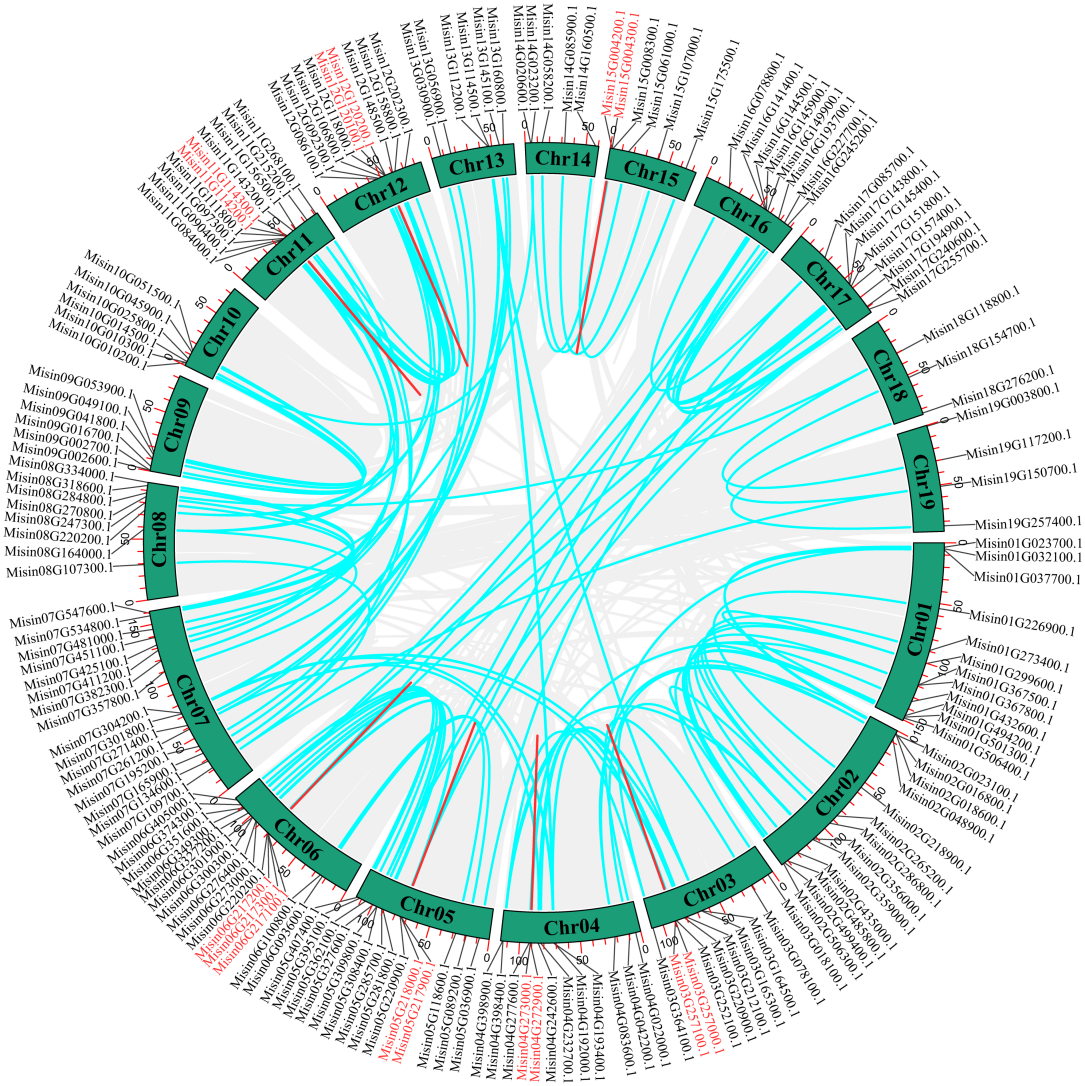
Schematic representations of the interchromosomal relationships of *M. sinensis NAC* genes. Gray lines indicate all synteny blocks in the genome, and the blue lines indicate segmental duplicates of *NAC* gene pairs, and the red lines indicate tandem duplications of *NAC* gene pairs.

The genomic locations of *M. sinensis NAC* are shown in Figure 3 (Supplementary Table S1). A total of 234 *NAC* genes were unevenly distributed on 19 chromosomes. The maximum number of *NAC* genes exhibited in Chromosome 7 (22, ~9.40%), while the least number of *NAC* genes (7, ~2.99%) were located on chromosome 10. Additionally, the distribution of *NAC* genes on chromosomes 10, 11, and 12 was relatively concentrated. There were 27 *NAC* genes just mapping to the scaffolds of *M. sinensis* genome. Tandem duplication is an essential source for the origin and evolution of multigene families. In this study, only nine pairs of tandem duplicate genes in *M. sinensis NAC* gene family were identified after the duplication events of *NAC* genes were examined, which were linked with the red line (Figure 4). There were 121 segmental duplication gene pairs identified in *M. sinensis NAC* gene family in all subpopulations. The results suggested that gene duplication, especially segmental duplication, may provide the primary driving force of evolution and may be possibly associated with the amplification of *M. sinensis NAC* gene family.

### Syntenic Analysis of *NAC* Genes Between *M. sinensis* and Several Related Plants

**Figure 5:**
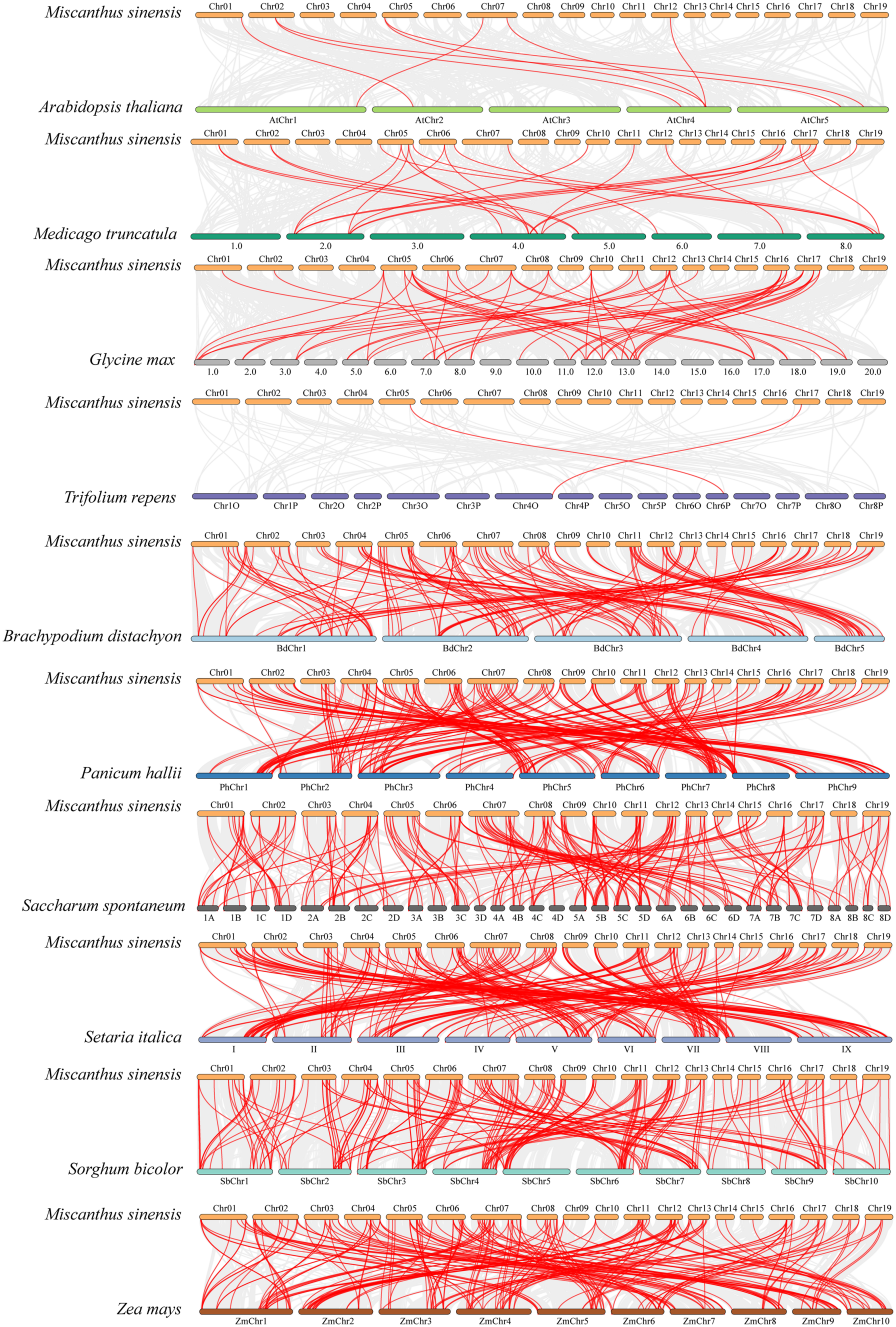
Synteny analysis of *NAC* genes between *M. sinensis* and 10 representative plant species. Gray lines in the background indicate the collinear blocks within the *M. sinensis* and other plant genomes, whereas the red lines highlight the syntenic *NAC* gene pairs.

In order to further explore the evolutionary relationship of *NAC* gene family in *M. sinensis*, 10 comparative syntenic maps consisting of four dicotyledonous plant (*A. thaliana*, *M. truncatula*, *G. max*, and *T. repens*) and six monocotyledonous plants (*B. distachyon*, *P. hallii*, *S. spontaneum*, *S. italic*, *S. bicolor*, and *Z. mays*) were constructed (Figure 5). The number of homologous pairs between *M. sinensis* and 10 other species was 2, 8, 25, 55, 148, 202, 205, 210, 211, and 215, respectively (Supplementary Table S3). By comparison, it was found that there are more homologous genes between *M. sinensis* and monocotyledons, indicating that these species were associated with the phylogenetic relationship.

### Expression Patterns of *NAC* Genes in Response to Various Abiotic Stress

**Figure 6:**
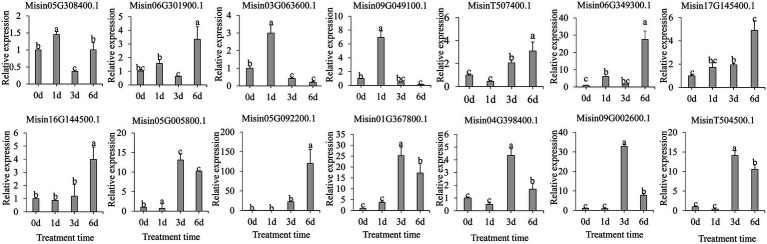
Expression profiles of 14 selected *M. sinensis NAC* genes in response to salinity treatment. Data were normalized to reference gene *Unigene33024,* and vertical bars indicate SD. No common letter above bar indicates a significant difference by least significant difference (LSD; *p* < 0.05).

It is evident that *SNAC* plays a vitally important role in the regulation of plant stress tolerance. To investigate the expression pattern of *NAC* genes under abiotic stress, 14 *M. sinensis NAC* genes were selected based on the phylogenetic tree in SNAC subgroup. Under salt treatment (Figure 6), most genes were shown to be significantly upregulated and obviously occurred at 1d (*Misin03G063600.1* and *Misin09G049100.1*), 3d (*Misin05G005800.1*, *Misin01G367800.1*, *Misin04G398400.1*, *Misin09G002600.1*, and *MisinT504500.1*), or 6d (*Misin06G301900.1*, *Misin06G349300.1*, *Misin17G145400.1*, *Misin16G144500.1*, and *Misin05G092200.1*), which indicates an emergency rescue for plant response to stress (Figure 6).

**Figure 7:**
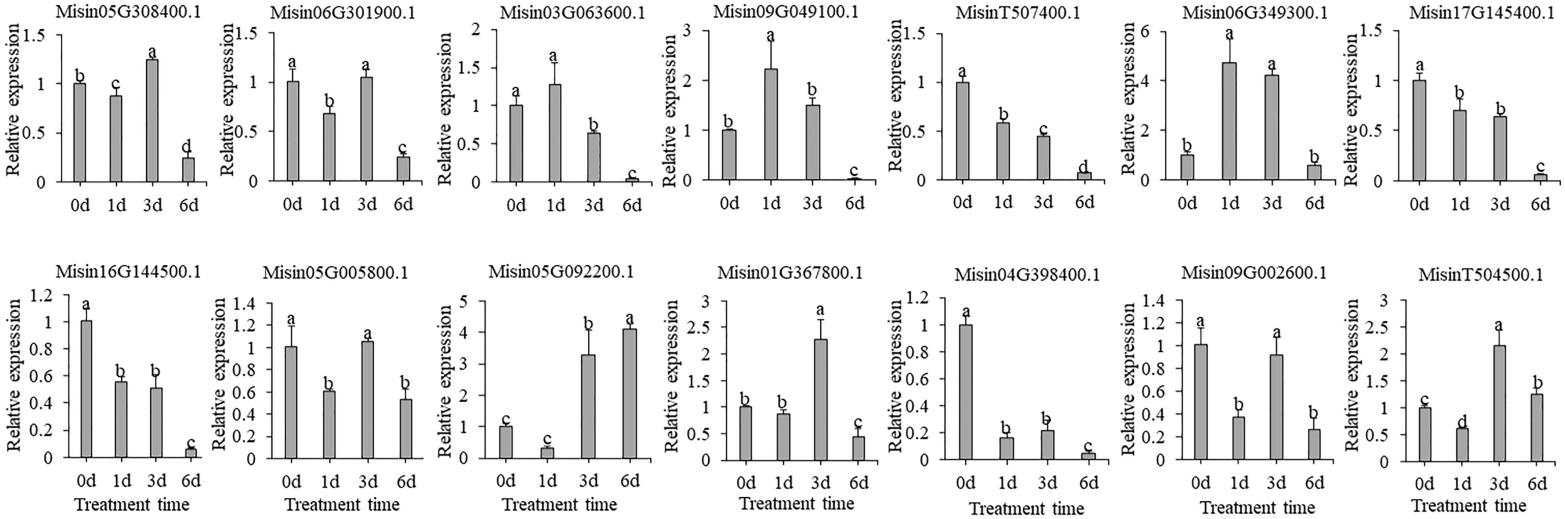
Expression profiles of 14 selected *M. sinensis NAC* genes in response to PEG treatment. Data were normalized to reference gene *Unigene33024,* and vertical bars indicate SD. No common letter above bar indicates a significant difference by least significant difference (LSD; *p* < 0.05).

In contrast, the gene expression profile was very different between drought and salt treatments. The expression of most selected candidate genes did not result in significant fold changes, and some genes were shown to be downregulated (*MisinT507400.1*, *Misin17G145400.1*, *Misin16G144500.1*, and *Misin04G398400.1*) after exposure to PEG treatment (Figure 7).

**Figure 8:**
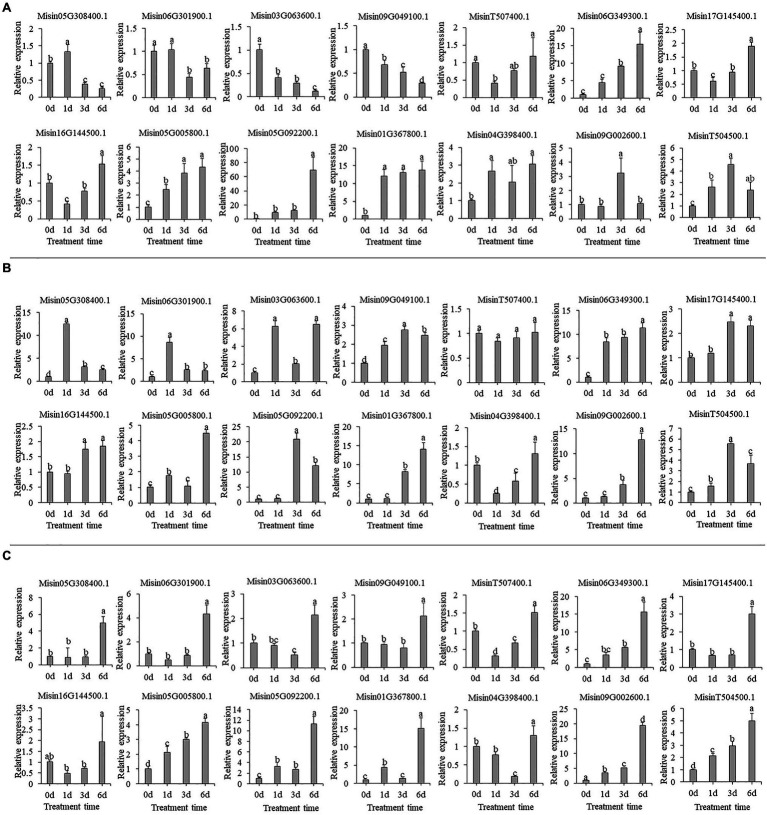
Expression profiles of 14 selected *M. sinensis NAC* genes in response to **(A)** Cr **(B)** Cd and **(C)** As treatment. Data were normalized to reference gene *Unigene33024,* and vertical bars indicate SD. No common letter above bar indicates a significant difference by least significant difference (LSD; *p* < 0.05).

Multiple *NAC* genes could be induced by heavy metal treatment. In Cr treatment, most genes had significantly increased expression, especially for *Misin06G349300.1*, *Misin05G092200.1*, and *Misin01G367800.1*. However, two *NAC* genes were downregulated during treatment time (Figure 8A). Consistently, there were also intense changes of gene expression under Cd treatment, and most genes upregulated continuously except for *Misin05G308400.1* and *Misin06G301900.1*, for which peak values of relative expression appeared at 1d (Figure 8B). A similar gene expression pattern was found during As treatment. For instance, four *NAC* genes (*Misin06G349300.1*, *Misin05G092200.1*, *Misin01G367800.1*, and *Misin09G002600.1*) were highly induced and showed continuously increasing expression (Figure 8C). To determine gene function prediction, the gene expression pattern could provide crucial information.

## Discussion

*Miscanthus genus* is one of the most promising non-food bioenergy crops belonging to the *Tribe Andropogoneae* (Poaceae), which contains many important C4 crops, including sugarcane (*S. officinarum* L.), sorghum (*S. bicolor* L.), and maize (*Z. mays* L.; Nie et al., 2016). However, owing to the complexity of *Miscanthus* genome, the lack of reliable genome information and unavailable genetic databases limited related research within the genus. Recently, chromosome-scale assembly of *Miscanthus lutarioriparius* genome (Miao et al., 2020), *Miscanthus floridulus* genome (Zhang et al., 2021), and *M. sinensis* genome (Mitros et al., 2020) was published, which would provide a valuable genetic resource for *Miscanthus* breeding improvement and molecular regulation network of stress resistance. The *NAC* gene family is a crucial plant-specific transcription factor family that is well known for its involvement in plant growth and developmental activities along with resistance to biotic and abiotic stresses (Wang et al., 2014; Dalman et al., 2017).

Attributing to the differences in genome size for each species, members of the *NAC* gene family identified in multiple plant species were varied. In *A. thaliana*, there were 117 *NAC* genes obtained from the genome database (Ooka et al., 2003), while 72 *NAC* genes have been identified in *Lolium perenne* (Nie et al., 2020), 80 in *F. tataricum* (Liu et al., 2019), 108 in *Dactylis glomerata* (Yang et al., 2021), 152 in *G. max* (Le et al., 2011), 151 in *Oryza sativa* (Nuruzzaman et al., 2010), 152 in *Z. mays* (Shiriga et al., 2014), and 288 in *T. aestivum* (Guérin et al., 2019). In this study, a total of 261 *NAC* genes were identified based on the *M. sinensis* genome (size of 1.88 G, Mitros et al., 2020), which is higher than most other plant species, indicating they contribute to the varied genome size (such as *A. thaliana*, 125 Mb; Korbinian et al., 2011): *L. perenne*, 2.04 Gb (Byrne et al., 2015); *F. tataricum*, 489.3 Mb (Zhang et al., 2017); *D. glomerata*, 1.78 Gb (Huang et al., 2020); *G. max*, 1.025 Gb (Shen et al., 2018); *O. sativa*, 466 Mb (Guillaume et al., 2003); and *T. aestivum*, 14.5 Gb (Appels et al., 2018). Continued gene duplication events occurred even after differentiation from their earliest ancestors. Besides, a collinearity analysis showed that there were nine groups of tandem duplication and 121 segmental duplication events in *M. sinensis NAC* gene family were identified. The duplication event of *M. sinensis* increases the genome size rather than increasing many *NAC* gene members, while tandem replication of *NAC* genes has been observed in many species. The result may be related to the expansion of long terminal repeat retrotransposons. Moreover, the structure of the genes was relatively conserved, while the length, MW, and pI of the *NAC* genes varied. The motifs of transcription factors are often related to DNA binding, protein interaction, and transcriptional activity (Liu et al., 2019). Owing to the function of *NAC* genes being predicted *via* alignment with model plants, such as *Arabidopsis*, the conserved motifs and gene structures may provide fundamental information for classification in *M. sinensis* (Nie et al., 2020).

Some NAC proteins have a TM motif in the C-terminus and show subcellular localization at the plasma membrane or endoplasmic reticulum. These proteins may be regulated by post-translational regulation under specific conditions (Seo et al., 2010; Kim et al., 2012; Lee et al., 2012). In this research, 12 *M. sinensis* NAC proteins were identified, which contained α-helical TM domain. In line with that, several species have membrane-bound NAC transcription factors, such as 13 identified in *Arabidopsis*, 13 in tomato (*Solanum Lycopersicum* L.), 11 in soybean, and five in rice, and most are induced by abiotic stresses (Kim et al., 2007; Le et al., 2011; Bhattacharjee et al., 2017). A previous study confirmed that the gene expression patterns can provide crucial information for determining gene function prediction (Zhou et al., 2018a). In this study, results showed that the NAP subgroup members *Misin05G092200.1* and *Misin01G367800.1* had increased expression under all five stress conditions, which was perhaps related to the abiotic stress resistance of *M. sinensis*. Interestingly, a previous study revealed that *NAC* genes in the NAP subgroup were involved in plant leaf senescence and floral morphogenesis, as well as associated to abscisic acid hormones regulating (Sablowski and Meyerowitz, 1998; Guo and Gan, 2006), which could be an important reason indicating stress resistance function of these kinds of genes. Besides, it was proved that AtSOG1ΔNAC (*AT1G25580*) was induced by salinity and the stability of SOG1 protein under salinity stress could be regulated by N-terminal NAC domain and the C-terminal (Mahapatra and Roy, 2019). Based on phylogenetic and conserved gene structure analysis, it was speculated that *Misin11G114300.1* (together with *AT1G25580*, which were assigned into ONAC003 subgroup) had possible relevance to salinity stress. *Arabidopsis* resistance to drought, high salinity, and abscisic acid was significantly enhanced through the overexpression of *AtNAC3* (Tran et al., 2004), which was similar to these homologous genes in *M. sinensis* from expression results. However, although *Misin05G308400.1* and *Misin06G301900.1* were duplicated genes within the same subgroup (AtNAC3), the expression pattern of *Misin05G308400.1* was different from that of *Misin06G301900.1* in salinity treatment, which might be caused by variation in gene regulation after duplication events. The result was consistent with a previous study in *F. tataricum* that showed the expression pattern between *FtNAC15* and *FtNAC38*. An *Arabidopsis* NAC transcription factor family gene (*AtNAP*) functions as a negative regulator *via* transcriptional repression of *AREB1* under salt stress (Seok et al., 2017). Expression of the *SNAC* gene *Misin09G002600.1* in *M. sinensis* significantly reached its peak value at 6d, which was over 100 times higher than the control in salinity stress. In general, these findings supported the view that the NAC proteins in SNAC subgroup actively participated in plant development and stress response (Takasaki et al., 2010, 2015), and these candidate genes with putative functions could be further validated in future.

In addition, a previous study had shown that *MsSND1* (annotated *Misin19G001800.1* in the genome belonging to the subgroup OsNAC7) is an NAC transcriptional master regulator orchestrating secondary cell wall biosynthesis in *Miscanthus* (Golfier et al., 2017), and the biosynthesis of lignin could directly reduce the entry of heavy metals into the root system (Cheng et al., 2014). Consequently, the predicted function of *Misin19G001800.1* may play a role in the tolerance of *M. sinensis* to heavy metals, which would provide a new insight to further verify the function of *M. sinensis NAC* genes.

## Conclusion

The release of *M. sinensis* genome provided a basic platform for the genome-wide investigation of NAC proteins. A total of 261 *M. sinensis NAC* genes were identified, and a comprehensive analysis of the gene family was presented in this study, including chromosomal distribution, conserved motif compositions, gene structure, and gene duplications. Results showed that gene length, MW, and pI of NAC family were varied, while gene structure and motifs were relatively conserved. Chromosomal mapping analysis found that the *M. sinensis NAC* genes were unevenly distributed on 19 *M. sinensis* chromosomes, and the interchromosomal evolutionary analysis showed that nine pairs of tandem duplicate genes and 121 segmental duplications were identified. The expression patterns of 14 genes from *M. sinensis* SNAC subgroup were analyzed under high salinity, PEG, and heavy metals, and multiple *NAC* genes could be induced by the treatment, indicating that abiotic stress-responsive NAC-type transcription factors play important roles for improving the stress tolerance of *M. sinensis*. However, further investigation of extensive functional characterization of the *NAC* gene family in *M. sinensis* is needed. Consistently, current results will provide very useful information for a follow-up study of *NAC* genes in stress-response and potential genetic improvement for better adaptivity in marginal land.

## Data Availability Statement

Publicly available data sets were analyzed in this study. These data can be found at: https://phytozome-next.jgi.doe.gov/info/Msinensis_v7_1.

## Author Contributions

XZ, GN, YP, and LH contributed to experiment design and funding acquisition. Material preparation and data collection were performed by ZY, JH, AL, and JC. DL, ZY, JH, GF, and XW analyzed data. The first draft of the manuscript was written by GN, ZY, SW, and JH. All authors commented on previous versions of the manuscript. All authors read and approved the final manuscript.

## Funding

This work was supported by China Agriculture Research System of MOF and MARA, Grassland Basic Resources Investigation Research in China (2017FY100602), and the National Natural Science Foundation of China (NSFC 31801432).

## Conflict of Interest

The authors declare that the research was conducted in the absence of any commercial or financial relationships that could be construed as a potential conflict of interest.

## Publisher’s Note

All claims expressed in this article are solely those of the authors and do not necessarily represent those of their affiliated organizations, or those of the publisher, the editors and the reviewers. Any product that may be evaluated in this article, or claim that may be made by its manufacturer, is not guaranteed or endorsed by the publisher.

## Footnotes

^1^ https://phytozome-next.jgi.doe.gov/info/Msinensis_v7_1

^2^ http://pfam.xfam.org/

^3^ http://pfam.xfam.org/search#tabview=tab1

^4^ https://www.ncbi.nlm.nih.gov/Structure/bwrpsb/bwrpsb.cgi

^5^ https://web.expasy.org/protparam/

^6^ http://linux1.softberry.com/berry.phtml

^7^ http://www.cbs.dtu.dk/services/TMHMM/

^8^ https://www.arabidopsis.org/

^9^ http://meme-suite.org/tools/meme

^10^ http://mg2c.iask.in/mg2c_v2.0/

^11^ https://github.com/CJ-Chen/TBtools

## Supplementary Material

Supplementary Figure S1 The sequence information for each motif.
