# Erratum: Genome-Wide Investigation of the NAC Transcription Factor Family in *Miscanthus sinensis* and Expression Analysis Under Various Abiotic Stress

**DOI:** 10.3389/fpls.2021.832799

**Published:** 2022-01-06

**Authors:** Frontiers Production Office

**Affiliations:** Frontiers Media SA, Lausanne, Switzerland

**Keywords:** *Miscanthus sinensis*, NAC transcription factor, gene family, abiotic stress, gene expression

Due to a production error, an incorrect version of the article was published. The article text has been copyedited to enhance language usage and comprehension.

The publisher apologizes for this mistake. The original version of this article has been updated.

## Publisher's Note

All claims expressed in this article are solely those of the authors and do not necessarily represent those of their affiliated organizations, or those of the publisher, the editors and the reviewers. Any product that may be evaluated in this article, or claim that may be made by its manufacturer, is not guaranteed or endorsed by the publisher.

