# Supplementary figures and images for: Genome-Wide Investigation of the NAC Transcription Factor Family in Miscanthus sinensis and Expression Analysis Under Various Abiotic Stresses
Source: Front Plant Sci. 2021 Nov 4;12:766550. doi: 10.3389/fpls.2021.766550 (PMC8600139; doi:10.3389/fpls.2021.766550)

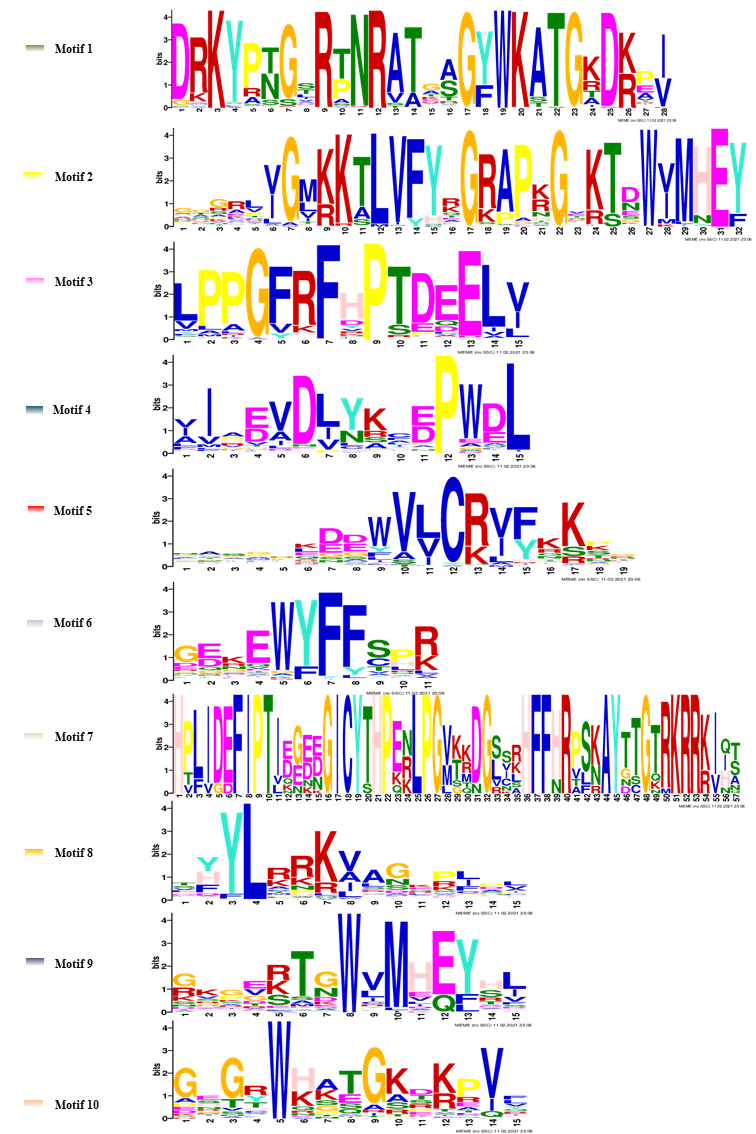

Supplement: Supplementary Figure S1 — The sequence information for each motif. [file Data_Sheet_1.zip › Supplementary Material/Figure S1.tif]
