# Supplementary material for: Genome-Wide Investigation of the NAC Transcription Factor Family in Miscanthus sinensis and Expression Analysis Under Various Abiotic Stresses
Source: Front Plant Sci. 2021 Nov 4;12:766550. doi: 10.3389/fpls.2021.766550 (PMC8600139; doi:10.3389/fpls.2021.766550)
Supplement: Supplementary Figure S1 — The sequence information for each motif. [file Data_Sheet_1.zip › Supplementary Material/Supplementary Material .doc]

# Supplementary Material

Table S1. The basic information of all identified *M. sinensis NAC* genes.

Table S2. Analysis of cis-acting element of *M. sinensis* *SNAC* genes.

Table S3. The number of homologous pairs between *M. sinensis* and other seven species.

Figure S1. The sequence information for each motif.
